# Correlation between subcutaneous adipose tissue of the head and body mass index in children and young adults aged 8–19 years: implications for functional neuroimaging

**DOI:** 10.3389/ebm.2024.10030

**Published:** 2024-02-08

**Authors:** Stacey L. Gorniak, Hao Meng, Saba Yazdekhasti, Luca Pollonini

**Affiliations:** ^1^ Department of Health and Human Performance, University of Houston, Houston, TX, United States; ^2^ Department of Engineering Technology, University of Houston, Houston, TX, United States; ^3^ Department of Electrical and Computer Engineering, University of Houston, Houston, TX, United States; ^4^ Department of Biomedical Engineering, University of Houston, Houston, TX, United States

**Keywords:** body fat, sex differences, EEG, fNIRS, obesity, tDCS, adolescents

## Abstract

High body mass index (BMI) is presumed to signify high amounts of fat (subcutaneous adipose tissue) distributed across the body. High amounts of fat co-occurring with increased BMI has been cited as a potential neuroimaging barrier. Presence of increased fat may result in high electrical impedance and increased light diffusion—resulting in low signal to noise ratios during electroencepholography (EEG), functional near-infrared spectroscopy (fNIRS), and transcranial direct current stimulation (tDCS) measurements. Examining if subcutaneous fat in the head increases with respect to total body fat percentage and BMI in school-aged children and adolescents is an essential next step in developing possible mathematical corrections for neuroimaging modalities. We hypothesized that percentage of subcutaneous adipose tissue in the head region would increase with respect to both total body fat percentage and BMI. Increased subcutaneous head fat percentage was associated with a positive linear relationship with BMI and a quadratic relationship with total body fat. The data indicate that participant age, sex, and adiposity should be considered in the development of model corrections for neuroimaging signal processing in school-aged children and adolescents. Strength of regression coefficients in our models differed from those in adults, indicating that age-specific models should be utilized.

## Impact statement

This project serves to provide justification for the development of signal to noise correction algorithms for neuroimaging modalities such as electroencepholography (EEG), functional near infrared spectroscopy (fNIRS), and transcranial direct current stimulation (tDCS). Noise in each of these neuroimaging modalities exists due to differences in subcutaneous adiposity, sex, and age across participants. Consideration and correction of these sources of noise will have a significant clinical impact on reported outcomes and life-long treatments plans for pediatric patients.

## Introduction

Increased body mass leading to overweight status or obesity is one of the most common health problems globally [1]. The World Health Organization (WHO) has recently estimated that more than 39 million school-aged children (aged 6–11 years) and 340 million adolescents (aged 12–19 years) are overweight or obese worldwide [2]. In the United States alone, over 20% of school-aged children and adolescents meet the criteria for obesity [3]. The definition of overweight is a Body Mass Index (BMI) ≥ 25 kg/m^2^ and the definition of obesity is BMI ≥ and 30 kg/m^2^, as per WHO guidelines [4]. High BMI is presumed to signify a large amount fat distributed across the body, whereas amount of body fat is measured via gold-standard techniques such as Dual-energy X-ray absorptiometry (DXA). Studies have shown that BMI differs from total body fat percentage across the lifespan [5, 6]. Specifically, it has been shown that BMI exhibits a non-linear relationship with body fat percentage that is impacted by both age and sex [5–7].

Despite the differences in adiposity measures, both high BMI and increased body fat are associated with the development of increased risk for cardiovascular diseases, metabolic disease (e.g., Type 2 Diabetes), musculoskeletal disease, cancer, and negative impacts to mental health across the lifespan [4, 8]. In recent years, increased subcutaneous adipose tissue has been cited as a barrier for non-invasive neuroimaging technologies, including electroencepholography (EEG), functional near infrared spectroscopy (fNIRS), and transcranial direct current stimulation (tDCS) [9, 10]. Recent interest in the impact of obesity on these neuroimaging technologies has manifested within the evidence base. In EEG [11–13] and tDCS [14, 15]-based research, the concern is that increased subcutaneous adipose tissue within the head may cause high electrical impedance and reduce the amplitude of signals detected through the derma during measurement. In fNIRS [16]-based research, the concern is that the increased subcutaneous adipose tissue will cause increased light diffusion from emitter optodes which will reduce the signal detected by the detector optodes during testing.

In each of these cases, the reduced signal strength to be detected during these evaluations is a major concern, leading to exclusion of participant or patient data due to low signal to noise ratios in individuals with increased amounts of adiposity. This exclusion is a form of phenotypic bias that disproportionally impacts underrepresented minorities, given the higher incidence of overweight and obesity in these populations [3, 17]. The presence of such phenotypic biases due to an inattention of adipose impacts on EEG, fNIRS, and tDCS signal to noise characteristics may reinforce and amplify gaps in assessment and treatment of significant life-long health conditions impacting neurological function and mental health in school-aged children and adolescents with increased adipose tissue, particularly in those individuals that belong to underrepresented minority groups [18].

As a countermeasure to these potential biases that can affect long-term health outcomes across the lifespan and disproportionally impacting underrepresented minority groups, adiposity should be considered as a potential confounder of cortical activity measurement occurring through the derma. Examining how subcutaneous adipose tissue in the head region increases with respect to percentage of total body fat and BMI in school-aged children and adolescents is an essential step in developing mathematical corrections to be implemented in these neuroimaging modalities, consisted with our prior findings in adults aged 20–89 years old [9].

Accordingly, the purpose of this project was to investigate how the percentage of subcutaneous adipose tissue in the head region in children and young adults aged 8–19 years is influenced by common adiposity metrics such as BMI and total body fat percentage. We hypothesized that percentage of subcutaneous adipose tissue in the head region would increase with respect to both percentage of total body fat percentage and BMI yet still carry influences of both age and sex, consistent with the adult models in our earlier work (Hypothesis 1). Consistent with our prior findings [9], we also expected total body fat to increase proportionally with BMI but that head fat percentage would increase modestly with BMI across the sample (Hypothesis 2). This information may be used by researchers and clinicians in improving neuroimaging signal to noise ratios for EEG, fNIRS, and tDCS data collected in pediatric populations as well as to avoid persistent exclusion biases due to physiologic characteristics (e.g., adiposity).

## Materials and methods

### Study participants

To evaluate the measures of interest for this study on a large scale, data from the National Health and Nutrition Examination Survey (NHANES) 2005–2006 were analyzed [19]. This particular data set included a body composition scanning protocol via DXA for a sample of individuals aged 8–69 living in the United States in addition to typical NHANES anthropomorphic data. Data from individuals in the 8–19 years old age groups were included in this project. Data from individuals aged 20–69 were assessed in a different project [9], as the sampling rates for individuals aged 20+ years were different from the 8–19 year-old age group (NHANES purposefully oversampled individuals aged ≤19 years). The data set in this study included a wide variety of BMI values. Specifically, BMI values within the data set ranged from 12.41 kg/m^2^ to 62.08 kg/m^2^. Data from 2,947 participants were included in this analysis (1,455 males and 1,492 females). Of the 2,947 participants within the NHANES 2005–2006 data set, 26 had data reported on only age and sex. A total of 2,921 participants with anthropometric data were assessed in the presented models. Demographics for the initial sample (*n* = 2,947) are located in Table 1. Characteristics of individuals in commonly considered BMI groups as per WHO anthropometry-based criteria [normal weight (NW), BMI ≤ 24.9 kg/m^2^; overweight (OW), BMI: 25.0–29.9 kg/m^2^; and the obese groups (OB), BMI ≥ 30.0 kg/m^2^] [4] are in Table 1. As this is a secondary analysis of the NHANES 2005–2006 data set (a de-identified data set), this project was considered exempt from review by the Institutional Review Board (IRB) at the University of Houston [20]. All NHANES participants provided written informed consent and/or assent in the original study.

**TABLE 1:** Age, anthropometry, BMI, total body fat %, and subcutaneous head fat % for each commonly referenced BMI range within the assessed data set.

	Normal weight (NW)		Overweight (OW)		Obese (OB)		Entire sample	
# Subjects	2,050		474		397		2,947	
Males:Females	1,052:998		214:260		176:221		1,455:1,492	
	Mean ± SD	[min–max]	Mean ± SD	[min–max]	Mean ± SD	[min–max]	Mean ± SD	[min–max]
Age (years)	13 ± 3	[8–19]	15 ± 3	[8–19]	16 ± 3	[8–19]	14 ± 3	[8–19]
Height (m)	1.57 ± 0.15	[1.12–2.00]	1.62 ± 0.12	[1.23–1.93]	1.65 ± 0.10	[1.38–1.97]	1.59 ± 0.15	[1.12–2.00]
Mass (kg)	49.85 ± 13.72	[20.00–88.20]	72.24 ± 11.31	[42.50–106.10]	97.21 ± 20.28	[61.20–215.3]	59.92 ± 22.19	[20.00–215.30]
BMI (kg/m^2^)	19.82 ± 2.77	[12.41–24.99]	27.27 ± 1.40	[25.02–29.99]	35.17 ± 5.37	[30.04–62.08]	23.12 ± 6.30	[12.41–62.08]
Body fat (%)	26.29 ± 7.23	[10.10–46.80]	35.25 ± 7.46	[16.2–50.00]	40.33 ± 6.19	[25.20–58.00]	29.60 ± 8.90	[10.10–58.00]
Head fat (%)	23.78 ± 0.42	[21.90–25.70]	24.13 ± 0.68	[22.60–26.60]	24.53 ± 0.76	[22.90–27.40]	23.94 ± 0.59	[21.90–27.40]

Range of data [minimum–maximum] are included for reference.

### Procedure

In the NHANES study, body mass (kg) and height (m) were measured for each participant. BMI was calculated as kg/m^2^ as per WHO guidelines [4]. Total and region-specific body composition of each participant was measured via Hologic DXA (Hologic QDR-4500A, Hologic, Inc., Bedford, MA, United States). DXA measures of interest for this study included % of total body fat and head region composition (including head fat %).

### Statistical analyses

Multiple regression analyses were performed via SPSS 25 (IBM Corporation, Armonk, NY, United States). All follow-up correlation analyses were performed via Minitab 17 (Minitab LLC, State College, PA, United States) to assess the strength of relationships among individual variables of interest. Consistent with our prior work, linear, quadratic, and logarithmic best fit regression models were assessed for measures of interest. In all models, age, BMI, total body fat %, and head fat % were considered continuous variables. *Sex* (two levels: coded as male = 0 and female = 1) was considered as a categorical variable. Anthropomorphic and DXA data were considered in imputation batches as recommended by the US Centers for Disease Prevention and Control (CDC) [21]. The data from the five NHANES imputation batches converged to the models presented in the results section. The model produced by imputation #1 is presented in this manuscript. After diagnosis using Cook’s D, no outliers were removed from the imputation #1 data set.

## Results

Means, standard deviations, and range values of age, body mass, height, BMI, total body fat %, and head fat % for all NHANES 2005–2006 study participants aged 8–19 years old are located in Table 1. Significant relationships among adiposity measures of interest (total body fat %, head fat %, and BMI) were found via regression (see Table 2). Age and sex significantly impacted all adiposity relationships. A logarithmic relationship between BMI and total body fat % was found. Linear relationships between head fat % and measures of full body adiposity (BMI and total body fat %) were noted. A quadratic model between head fat % and total body fat is also reported here; the quadratic model provided a modest improvement in R^2^ value as compared to the linear model.

**TABLE 2:** Regression analyses output.

Factor	Coefficient	t-value	*p*-value
Log(BMI) and total body fat regression model (*r* = 0.8476, adjusted *r* ^2^ = 71.84%)			
Age (years)	−1.0262	−35.01	<0.001
log_10_(BMI) [log_10_(kg/m^2^)]	62.8960	70.07	<0.001
Sex	7.7910	44.13	<0.001
Regression constant	−44.7900	−40.56	<0.001
Total body fat and head fat linear regression model (*r* = 0.6939, adjusted *r* ^2^ = 48.16%)			
Age (years)	−0.0544	−22.73	<0.001
Total body fat (%)	0.0456	44.79	<0.001
Sex	−0.4571	−25.27	<0.001
Regression constant	23.5743	522.98	<0.001
Total body fat and head fat quadratic regression model (*r* = 0.7269, adjusted *r* ^2^ = 52.84%)			
Age (years)	−0.0609	−26.34	<0.001
Total body fat (%)	−0.0492	−8.66	<0.001
[Total body fat (%)]^2^	0.0015	16.94	<0.001
Sex	−0.4026	−22.93	<0.001
Regression constant	24.9762	267.81	<0.001
BMI and head fat regression model (*r* = 0.7056, adjusted *r* ^2^ = 49.78%)			
Age (years)	−0.1069	−41.99	<0.001
BMI (kg/m^2^)	0.0624	46.46	<0.001
Sex	−0.1125	−7.22	<0.001
Regression constant	24.0467	622.37	<0.001

**FIGURE 1:**
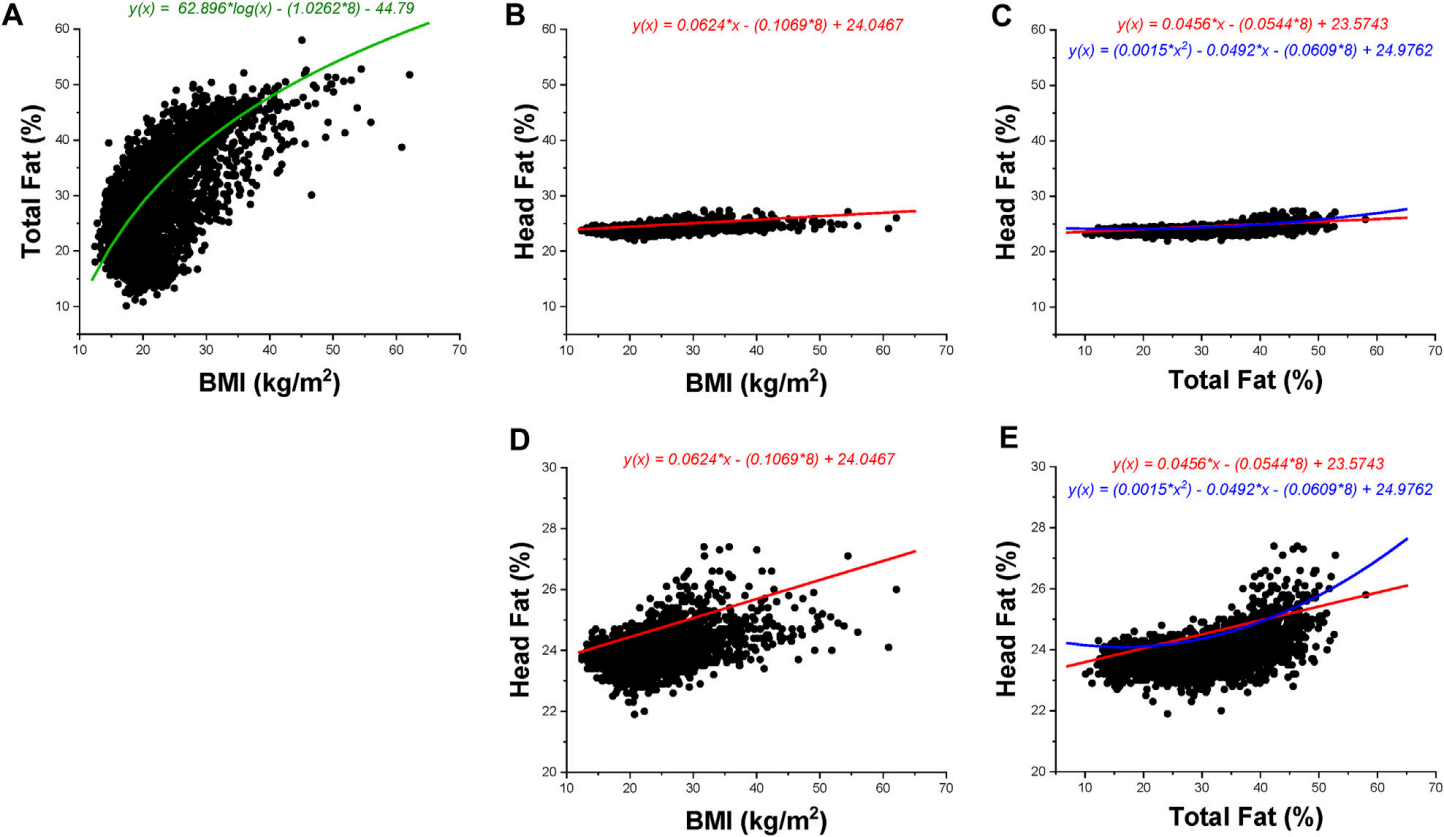
BMI, total body fat percentage, and head fat percentage data are presented for children aged 8–19 years. Black dots represent data points; the color-coded lines and corresponding color-coded equations are regression fits as per Table 2. Green indicates a logarithmic regression fit, red indicates a linear regression fit, and blue indicates a quadratic regression fit. All presented regression fits include age = 8 years (minimum age included in the data set) in the equation. The regression fit in **(A)** includes the sex indicator variable (1 = female). **(A–C)** Utilize the same axes values to show magnitude changes across the same scale; **(D–E)** illustrate data at a magnified scale. **(A)**: Total body fat percentage vs. BMI. **(B)**: Head fat percentage versus BMI (full scale). **(C)**: Head fat percentage versus total body fat percentage (full scale). **(D)**: Head fat percentage versus BMI (magnified scale). **(E)**: Head fat percentage versus total body fat percentage (magnified scale).

**FIGURE 2:**
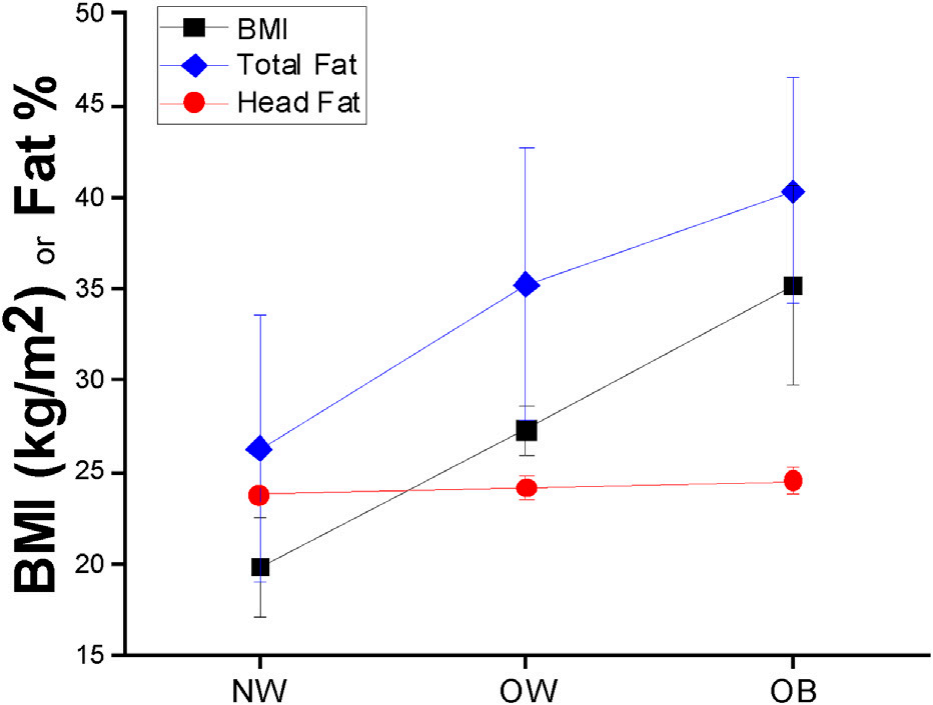
BMI (black squares), total body fat percentage (blue diamonds), and head fat percentage (red circles) mean ± SD values across BMI groups in children aged 8–19 years.

The correlation between log_10_(BMI) and total body fat % was found to be strong (*r* = 0.626), concurrent with moderate linear positive correlations between head fat % and general adiposity (*r* = 0.432 for head fat % vs. BMI; and *r* = 0.522 for head fat % vs. total body fat %). Figures 1A–E illustrates scatterplots of relationships among total body fat %, heat fat %, and BMI. Total body fat % strongly increased with BMI (an increase of 53.43% of total body fat was found across the sample groups); however, the increase in head fat % with BMI was much smaller, at only 3.16% across the sample groups. Differences in adiposity measures across WHO BMI categories are found in Figure 2. The Eqs. 1–4 describing the significant relationships among adiposity measures of interest found in Table 2 are below:

With respect to the relationship between BMI and total body fat, the logarithmic equation describing their relationship is:

$$Total\;Fat\;\left(\%\right)=-44.79-1.0262*Age\left(years\right)+62.896*\log_{10}\left(BMI\left(kg/m^{2}\right)\right)+7.791*Sex$$ (1)

where age is in years, BMI is in kg/m^2^, total body fat is in %, and sex is coded as male = 0 and female = 1.

With respect to the relationship between total body fat % and subcutaneous head fat %, the equation describing their linear relationship is:

$$Head\;Fat\;\left(\%\right)=23.5743-0.0544*Age\left(years\right)+0.0456*Total\;Fat\;\left(\%\right)-0.4571*Sex$$ (2)

where age is in years, total body fat is in %, subcutaneous head fat is in %, and sex is coded as male = 0 and female = 1.

With respect to the relationship between total body fat % and subcutaneous head fat %, the equation describing their quadratic relationship is:

$$Head\;Fat\;\left(\%\right)=24.9762-0.0609*Age\left(years\right)-0.0492*Total\;Fat\;\left(\%\right)+0.0015*\left({Total\;Fat\;\left(\%\right)}^{2}\right)-0.4026*Sex$$ (3)

where age is in years, total body fat is in %, subcutaneous head fat is in %, and sex is coded as male = 0 and female = 1.

With respect to the relationship between BMI and subcutaneous head fat %, the linear equation describing their relationship is:

$$Head\;Fat\;\left(\%\right)=24.0467-0.1069*Age\left(years\right)+0.0624*BMI\;\left(kg/m^{2}\right)-0.1125*Sex$$ (4)

where age is in years, BMI is in kg/m^2^, subcutaneous head fat is in %, and sex is coded as male = 0 and female = 1.

## Discussion

The purpose of this manuscript was to investigate how the percentage of subcutaneous adipose tissue in the head region in children and young adults aged 8–19 years is influenced by commonly used adiposity metrics (e.g., BMI). Consistent with our prior work in adults and older adults, we hypothesized that percentage of subcutaneous adipose tissue in the head region would increase with respect to both total body fat percentage and BMI yet still carry influences of both age and sex (Hypothesis 1). Also in line with our prior findings [9], we expected total body fat to increase proportionally with BMI but that head fat percentage would increase modestly with BMI across the sample (Hypothesis 2).

With respect to Hypothesis 1, a significant increase in subcutaneous head fat percentage was found concurrent with increased BMI (*r* = 0.432) and total body fat percentage (*r* = 0.522). These correlation values were lower than similar comparisons in adults and older adults (*r* = 0.668 and *r* = 0.62, respectively) [9]. Relationships among measures of interest were also influenced by age and sex. In particular, age was found to have a stronger influence on the current data set (children and young adults aged 8–19 years, |t-values| ≥ 22.73) as compared to adults (|t-values| ≥ 4.50) [9]. In contrast, the magnitude of the influence of sex differences appears lower in children and young adults aged 8–19 years (|t-value| range = 7.22–44.13) as compared to adults (|t-value| range = 14.55–92.84) [9]. The relationship between total body fat percentage and head fat percentage was found to be strongest using a quadratic model, which offered a modest improvement in accounting for model variability (+4.68% as compared to the linear model), whereas adult and older adult data exhibited linear trends [9]. This indicates that head fat percentage appears to become linearly related to total body fat percentage as individuals move into adulthood.

In consideration of Hypothesis 2, head fat percentage exhibited a small increase with BMI as compared to total body fat percentage (3.16% versus 53.43%, respectively). This small increase is similar to our findings in adult models (7.8% and 56.5%, respectively) [9] and findings reported from MRI work [22]. Based on these findings, it is probable that an increased amount of subcutaneous adipose tissue within the head region interferes with neuroimaging tools that measure physiological signals through the derma—confirming concerns that have emerged in the evidence base [9–16]. These tools are particularly susceptible to subcutaneous adipose tissue given the increased electrical resistance of adipose tissue [23] as well as inconsistencies in light propagation across adipose tissue [24]. Consideration of subcutaneous fat as a confounder is important given emerging evidence altered cortical function in persons with obesity (e.g., impaired motor cortex plasticity), which may have life-long impacts [25]. The data in this manuscript indicate that age, sex, and adiposity measures should be considered in the development of model corrections for neuroimaging across the lifespan. Despite similarities in the regression factors in the models generated for children and adults, there are differences in the strengths of relationships within the developed models (e.g., magnitude of regression coefficients, t-values, etc.). Use of age-range specific models is highly encouraged (models developed for ages 8–19 years for children and young adults versus models developed for ages 20–89 years for adults and older adults). We acknowledge at this time that models are not yet developed for children aged 7 years and younger—this is a future direction of our work.

Given the similarity of *r* ^2^ values across the models developed from this data set, use of either BMI or total body fat percentage in model corrections with respect to head fat percentage as a source of noise in neuroimaging is recommended for children aged 8–19 years. As BMI only requires basic anthropometric data such as height and mass, it is likely that BMI will be easier for the vast majority of investigators to use for model corrections. Use of total body fat in model corrections is encouraged for investigators who have access to DXA technology for more comprehensive physiological characterization in their model corrections. By accounting for the influence of subcutaneous adipose tissue of the head in neuroimaging studies, a more inclusive approach to neuroscience is permitted at the participant level. Current practices include discarding data with poor signal to noise ratios—an issue that may be caused by increased subcutaneous adipose tissue under the derma of the head. The equations developed in this manuscript may be used to counter this phenotypic bias across the lifespan, which may have a significant impact on reported outcomes in pediatric populations and life-long treatments plans for pediatric patients [17].

We acknowledge that signal to noise corrections with respect to subcutaneous head fat should be explored for individual measurement types (e.g., EEG, fNIRS, and tDCS) and manufacturers for the 8–19 years age range. The development of equations for specific equipment and manufacturers are outside of the scope of this project. The data in this project serve to provide justification in support of recent reports in the evidence base [11–16] for the practice of developing signal to noise correction algorithms due to adiposity measures, sex, and age.

## Data Availability

Publicly available datasets were analyzed in this study. This data can be found here: https://www.cdc.gov/nchs/nhanes/index.htm.
